# Itaconate or how I learned to stop avoiding the study of immunometabolism

**DOI:** 10.1371/journal.ppat.1010361

**Published:** 2022-03-24

**Authors:** Carolina Coelho

**Affiliations:** 1 MRC Centre for Medical Mycology at University of Exeter, Exeter, United Kingdom; 2 The Institute of Biomedical and Clinical Science, College of Medicine and Health, University of Exeter, Exeter, United Kingdom; University of Maryland, Baltimore, UNITED STATES

## Introduction to itaconate

Itaconate is a mitochondrial metabolite, produced in high amounts by macrophages and monocytes of mice and humans upon activation by several inflammatory stimuli. In 2013, the identity of the itaconate-producing enzyme was revealed as aconitate decarboxylase 1, encoded by the gene *Acod1* (previously known as immune-responsive gene 1, *Irg1*). This clear linkage of production of a mitochondrial metabolite in response to inflammatory signalling immediately raised a flurry of interest. Indeed, itaconate has significant immunomodulatory properties and may pave the way for new immunomodulatory drugs. Presented here are several aspects of itaconate biology and a discussion of future research avenues.

## How is itaconate synthesised and transported?

**Fig 1:**
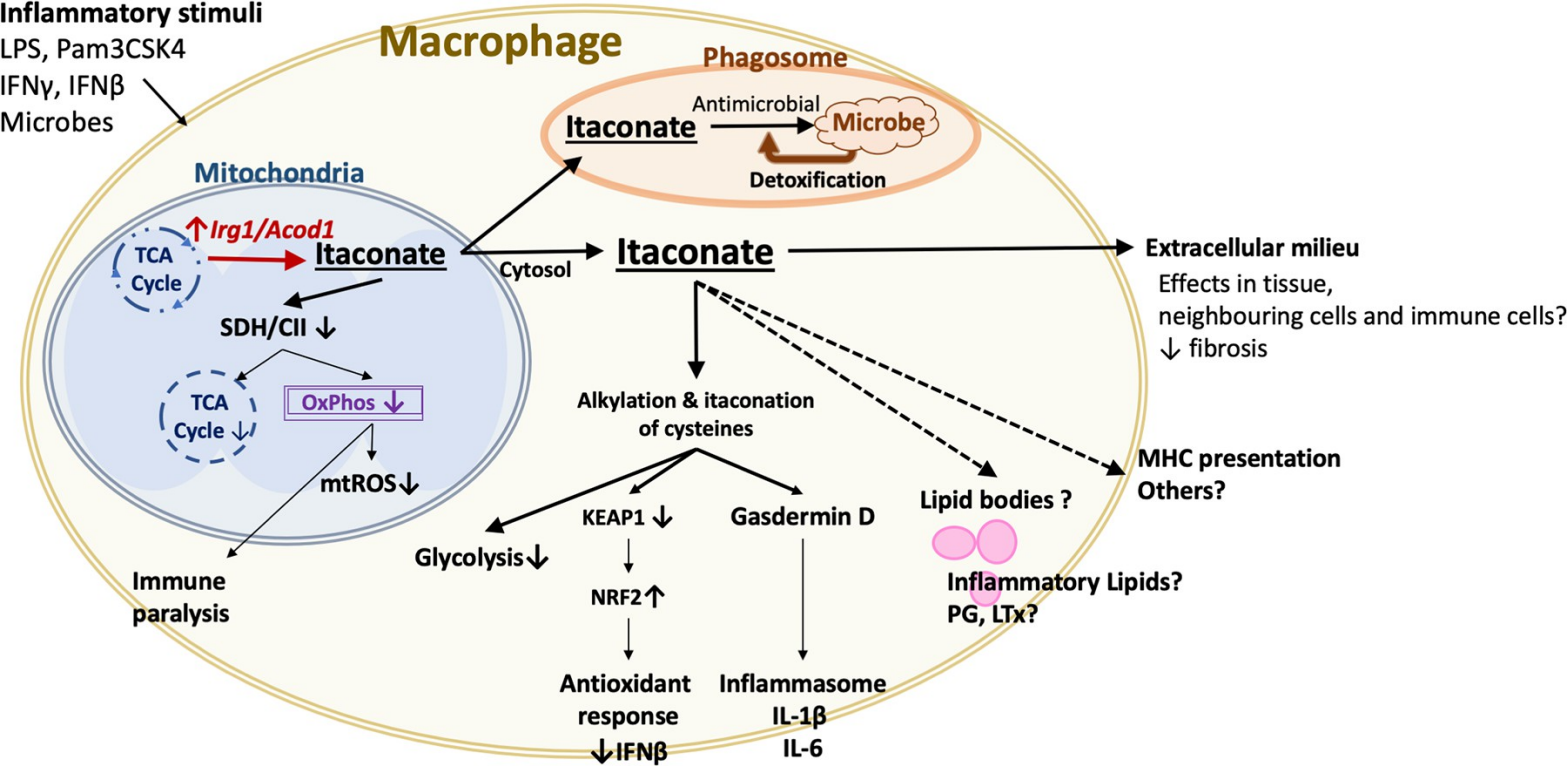
Itaconate modifies immune responses by controlling metabolism and posttranscriptionally regulating immune cascades, which is posited to prevent excessive tissue damage. IFNβ, interferon beta; IFNγ, interferon gamma; IL, interleukin; *Acod1/Irg1*: aconitate decarboxylase 1/immune-responsive gene 1; KEAP1, Kelch-like ECH-associated protein 1; LPS, lipopolysaccharide; LTx, leukotriene; MHC, major histocompatibility complex; mtROS, mitochondria-derived reactive oxygen species; NRF2, nuclear factor erythroid 2–related factor 2; OxPhos, oxidative phosphorylation; PG, prostaglandin; SDH/CII, succinate dehydrogenase and complex II of the respiratory chain; TCA cycle, tricarboxylic acid cycle.

Upon inflammatory stimuli, including microbial components like lipopolysaccharide (LPS) and fungal cell wall sugars as well as several interferon cytokines, macrophages up-regulate *Acod1*, the gene encoding aconitate decarboxylase 1 (or *cis*-aconitate decarboxylase). This mitochondrial enzyme (Fig 1) uses *cis*-aconitate from the citric acid cycle to synthesise itaconate. Following synthesis in the mitochondria, humans and other mammals can detoxify this metabolite with mitochondrial enzymes that convert itaconate into acetyl-CoA and pyruvate [1], but this metabolite does accumulate in high amounts for several hours after inflammatory stimuli in vitro [2]. Current evidence suggests that a pool of itaconate is exported to the cytosol, presumably by mitochondrial 2-oxoglutarate/malate carrier protein, although transporter specificity and redundancy are yet to be determined [3]. Itaconate is likely transported from the cytosol into the phagosome: Using gene-engineered bacterial sensors, itaconate was detected in the phagosomal compartment [4], presumably to exert antimicrobial activity. Similarly, addition of exogenous itaconate reverts many of the consequences of genetic deletion of *Acod1*, suggesting that transport into cells is efficient. A more detailed knowledge of itaconate transport within intracellular compartments and into neighbouring cells is urgently needed, particularly when one considers that itaconate may affect neighbouring cells in tissues (discussed below).

## Is itaconate antimicrobial?

Toxicity of itaconate to microbial pathogens is only observed at millimolar concentrations since successful pathogens have evolved to detoxify, tolerate, and even commandeer itaconate for their benefit.

The role of itaconate in microbial lifestyle and microbe’s response to stress is unknown. Production of itaconate is widespread in nature, produced by several fungi, including some *Aspergillus* species, and bacteria, such as *Bacillus subtilis* [5], giving these and other microbes ample opportunity to evolve the capacity to detoxify itaconate. An estimated 11% of all bacteria, including human pathogens such as *Yersinia pestis* and *Pseudomonas aeruginosa*, possess homologues of itaconate detoxifying genes, with conserved mechanisms producing acetyl-CoA and pyruvate [6,7]. Itaconate can be used as fuel by microbes, and some pathogens have even evolved to take advantage of itaconate production by the host. For example, *P*. *aeruginosa* isolates from cystic fibrosis patients are more likely to preferentially use itaconate as a carbon source [8].

Itaconate may synergise with other antimicrobial molecules and nutritional restrictions in the host phagosomal milieu to exert some antimicrobial effects. A very recent report showed that itaconate antimicrobial activity is potentiated by low pH, suggesting that within the phagosome, itaconate may become a significant microbicide [9]. However, the current data support that direct antimicrobial effects are not a major function of immune-derived itaconate.

Still, genetic deletion of *Acod1* increases mortality of mice infected by *Mycobacterium tuberculosis* and increases bacterial burden in mice infected with *Brucella* and *Salmonella* [10–13], likely due to a combination of direct and indirect effects in the host and in the pathogen. This model of coordinated action of itaconate with other immunoregulatory and antimicrobial factors is supported by findings that the combined actions of *Acod1* and 5 other immune genes, *Nos2*, *Cybb*, *Irgm1*, *Irgm3*, and *Casp4*, coordinate interferon gamma (IFNγ)-induced killing of *Legionella* in bone marrow–derived macrophages [14]. Given that both itaconate and nitric oxide (NO) [15,16] have important immunoregulatory effects on the host cell, including at the metabolic level, it is complex to dissect the immunomodulatory versus direct antimicrobial effects and requires the capability to precisely and reliably manipulate targets on each partner of the host–pathogen interaction.

### How does itaconate act as an immunomodulator?

The general framework is that itaconate acts as a negative regulator of innate immunity to limit host tissue damage, with some notable exceptions. The detailed molecular mechanisms of how itaconate exerts these effects are a very active area of research, with multiple mechanisms uncovered. Early on, a zebrafish (zebra danio) model of *Salmonella* infection showed the homologue of *Acod1* to be induced in macrophage lineage cells, in a manner dependent on the Jak/Stat pathway and glucocorticoid pathway [17]. Expression of the zebrafish (zebra danio) homologue of *Acod1* leads to mitochondrial reactive oxygen species (ROS) production in this model.

First, itaconate acts on mitochondria by competitive inhibition of succinate dehydrogenase (SDH) [18]. SDH is part of both the tricarboxylic acid cycle and the respiratory chain (complex II); thus, itaconate simultaneously interrupts the tricarboxylic acid cycle and reduces SDH-dependent oxygen consumption [16,19]. This results in succinate accumulation and a direct reduction of mitochondrial ROS (in contrast to the zebrafish (zebra danio) model). From the mitochondrial matrix, itaconate is transported into the cytosol where it can regulate metabolism at the glycolysis step, inhibiting key enzymes in this pathway to decrease glycolysis [20]. Thus, itaconate mediates several of the metabolic changes during inflammation; these direct effects are observed in most in vitro inflammatory models. Whether this occurs in vivo, and how it may be influenced by tissue microenvironment during acute inflammation and resolution stages, is still to be fully demonstrated: In a mouse model of pulmonary fibrosis, tissue-resident macrophages showed decreased oxygen consumption rate (OCR) in the absence of itaconate [21], in contrast to what is predicted by in vitro models.

Once in the cytosol (Fig 1), itaconate acts by directly modifying proteins via alkylation of cysteines [3]. In particular, alkylation of Kelch-like ECH-associated protein 1 (KEAP1) releases nuclear factor erythroid 2–related factor 2 (NRF2) [3], a potent antioxidant regulator, which then activates a second pathway to reduce cellular ROS.

Itaconate shows a lasting anti-inflammatory effect after LPS stimulation, generally decreasing levels of inflammatory cytokines interleukin (IL)-6, IL-1β, and via a negative feedback loop with interferon beta (IFNβ) [3]. Itaconate controls monocyte priming and immune paralysis after LPS treatment through an SDH-dependent mechanism in a model of human endotoxemia [22]. In other models, such as delayed inflammasome priming in murine bone marrow–derived macrophages, itaconate controls inflammasome activation via a novel posttranslational modification: itaconation [23]. Additional regulatory mechanisms of itaconate are still being uncovered. For example, itaconate controls the number of lipids bodies of macrophages after mycobacteria challenge [11]. Control of lipid bodies and lipid metabolism is likely a major regulatory pathway of itaconate [24,25], since lipid bodies are precursors for important inflammatory molecules such as prostaglandin and leukotrienes [26].

### How does itaconate regulate immunity at the tissue level?

Itaconate produced by myeloid cells can directly affect neighbouring cells. In a murine model of bleomycin-induced pulmonary fibrosis, itaconate produced by alveolar macrophages improved the healing pattern of lung fibroblasts; exogenous addition of itaconate influenced fibroblast healing pattern to ameliorate lung function [21]. Itaconate may also be produced by cells other than myeloid cells. Neurons were found to produce itaconate after challenged with Zika virus in vitro [27]. Curiously, neurons are able to take up exogenous itaconate, and itaconate application improved neurological function upon reperfusion injury [28]. Thus, itaconate can control tissue function by acting on nonmyeloid cells.

In mouse models of mycobacterial infections, deletion of *Acod1* leads to differential recruitment of immune cells in the lung, such as an increase in neutrophils [10] and in lymphocytes [11]. However, it is still unclear which immune signal is influencing this differential recruitment and whether itaconate may influence the development of adaptive immunity. Thus far, a single study observed that itaconate did not affect protective immunity by the Bacillus Calmette–Guérin (BCG) vaccine strain [11]. This observation fits current models at a conceptual level, as an attenuated strain BCG vaccine strain will not trigger excessive inflammation, but it is very intriguing at a mechanistic level, i.e., at which point is there enough inflammation to activate itaconate and through which cellular mechanisms? Is it possible that in vivo there is a need for tissue damage, and danger associated molecular patterns, to trigger production of itaconate? Thus, further studies understanding impact of itaconate in tissue homeostasis and influence on adaptive immunity are urgently required.

### Are analogues of itaconate potential immunomodulatory therapies?

In human studies, low levels of itaconate in plasma are associated with excessive inflammation in rheumatoid arthritis [28] and during Coronavirus Disease 2019 (COVID-19) [29], which strongly supports that supplement of itaconate and its analogues would be useful in clinic.

Currently, several analogues of itaconate show potent immunomodulatory properties. However, while these mimic several of itaconate functions, they show important differences from endogenously produced itaconate [25,29,30]. For example, analogues of itaconate prevents cycloxigenase-2 expression and production of several prostaglandin species in response to a Toll-like receptor (TLR)1/2 agonist Pam3CSK4, a possibly clinically useful anti-inflammatory effect, an effect not replicated by endogenous itaconate [25]. This is attributed to different reactivity of these analogues to act as cysteine modifiers and electrophilic molecules. Itaconate analogues showed beneficial effects in mouse models of psoriasis [31] and others autoimmune diseases. Interestingly, both dimethyl fumarate, a compound in clinical use to control psoriasis, and itaconate analogues were able to decrease prostaglandin expression in macrophages [25], suggesting some mechanistic and clinical overlap of itaconate analogues to existing therapies.

Overall, itaconate, and the immunobiology under its control, is revealing a wealth of knowledge on delicate equilibrium required of a successful immune response. While considerable work is still needed to fully understand these cascades, this knowledge holds great potential to improve our management of infectious and immune diseases.
